# Dapagliflozin reduces epicardial adipose tissue in patients with heart failure and type 2 diabetes

**DOI:** 10.1111/dom.70164

**Published:** 2025-10-06

**Authors:** Mohmmad Alghamdi, Adel Dihoum, Khalid Hakami, Atanu Bhattacharjee, Alexander J. M. Brown, Jagdeep Singh, Shaween Altalabany, Chim Lang, Ify Mordi, Faisel Khan

**Affiliations:** ^1^ Division of Cardiovascular Research University of Dundee Dundee UK; ^2^ College of Applied Medical Sciences Al‐Jouf University Sakaka Saudi Arabia; ^3^ Population Health and Genomics University of Dundee Dundee UK; ^4^ Department of Cardiology NHS Fife UK; ^5^ Department of Cardiac Imaging Erbil Cardiac Center Erbil Iraq

**Keywords:** cardiovascular disease, dapagliflozin, effectiveness, heart failure, type 2 diabetes

## Abstract

**Background:**

Epicardial adipose tissue (EAT) has a contributory role in the progression of heart failure. We tested whether dapagliflozin reduces EAT in adults with type 2 diabetes (T2D) and heart failure and explored links with systemic inflammation and cardiac structure.

**Methods:**

This analysis is based on pooled data from two phase 2, single‐centre, double‐blind, placebo‐controlled randomised trials (REFORM and DAPA‐LVH) conducted in Scotland. Exactly 122 participants with T2D and stage B or C heart failure were randomised to dapagliflozin 10 mg once daily or placebo for 12 months. Cardiac magnetic resonance imaging (CMR) was used to assess EAT. At baseline and follow‐up, the inflammatory markers TNF, IL‐1, IL‐6, IL‐10, and CRP were measured.

**Results:**

At baseline, obesity was common (75% with BMI ≥30 kg/m^2^) and heart‐failure phenotypes were balanced (HFpEF 51%, HFrEF 49%). After 12 months, dapagliflozin significantly reduced EAT independently of changes in BMI (−1.16 ± 0.18 vs. +0.36 ± 0.19 cm^2^, *p* < 0.001), BMI (−1.17 ± 0.16 vs. −0.18 ± 0.17 kg/m^2^, *p* < 0.001), and left ventricular mass (−3.53 ± 1.77 vs. +1.57 ± 1.83 g, *p* = 0.048) compared with placebo.

**Conclusion:**

Dapagliflozin shrinks EAT and LV mass independently of BMI in T2D patients with stage B/C heart failure, supporting EAT as a modifiable target of SGLT2 inhibition. The absence of parallel changes in systemic inflammation suggests primarily local mechanisms.

## 1 INTRODUCTION

Heart failure (HF) and diabetes are chronic conditions with a significant contribution to global morbidity and mortality.^1^ Together, these conditions lead to higher morbidity, mortality, and poorer outcomes compared with either condition alone.^2^ Recent studies confirmed the importance of epicardial adipose tissue (EAT) in the aggravation of cardiovascular diseases.^3^ EAT is a local visceral fat depot, located between the myocardium and visceral pericardium, and consists of a large population of energy‐storing adipocytes in addition to inflammatory, immune, and stromovascular cells.^4^, ^5^ EAT, under normal conditions, is an active endocrine organ that secretes bioactive molecules, including the heart‐protective anti‐inflammatory IL‐10. In pathological conditions, however, EAT becomes expanded, hypoxic, and infiltrated by macrophages, thus leading to chronic inflammation.^6^, ^7^ This results in increased production of proinflammatory adipokines, such as IL‐6, TNF, leptin, and resistin, leading to inflammation and deleterious effects on the heart and coronary arteries.^8^

EAT plays an important contribution to the pathophysiology of both heart failure with preserved ejection fraction (HFpEF) and heart failure with reduced ejection fraction (HFrEF).^9^, ^10^ EAT accumulation has been consistently associated with diastolic dysfunction, left ventricular hypertrophy, and atrial dilatation in HFpEF.^9^, ^11^, ^12^ Also, EAT may apply a pericardial restraint effect, leading to mechanistic compression of the myocardium and increasing cardiac filling pressures.^10^ The role of EAT in HFrEF has been studied less extensively; however, it may play a role in advancing the complication of heart failure across the spectrum of ejection fraction.^13^ Interestingly, EAT has a contrasting role in HFrEF compared with HFpEF. Most studies show that patients with HFrEF have lesser EAT volumes than those with HFpEF.^5^, ^14^ In contrast to HFpEF, lesser amounts of EAT are correlated with a worse prognosis in HFrEF.^15^, ^16^

Sodium–glucose cotransporter‐2 inhibitors (SGLT2i) are effective for glycaemic control and reduce atherosclerotic events, heart failure hospitalisations, cardiovascular mortality, and the progression of chronic kidney disease. Large clinical trials, such as EMPA‐REG OUTCOME, CANVAS PROGRAM, and DECLARE‐TIMI 58, have confirmed cardiovascular benefits in T2D patients with cardiovascular disease.^17^, ^18^, ^19^ More recent trials, including DAPA‐HF, DELIVER, EMPEROS Reduced and Preserved, have further demonstrated that these benefits extend to patients with heart failure regardless of diabetes status.^20^, ^21^, ^22^ The mechanisms of benefit of SGLT2i remain unclear but may be related in part to beneficial cardiac remodelling. The REFORM trial found no LV remodelling reversal but showed benefits like lower diastolic BP, reduced diuretic need, and increased haemoglobin.^23^ The DAPA‐LVH trial showed a reduction in left ventricular mass, lower blood pressure, weight loss, visceral and subcutaneous adipose tissue, and C‐reactive protein (CRP) levels.^24^, ^25^ These findings highlight SGLT2i as key for cardiovascular benefits in T2D and cardiovascular disease. Multiple meta‐analyses have demonstrated significant reductions in EAT with SGLT2 inhibitor therapy in patients with diabetes and obesity.^26^, ^27^, ^28^ Moreover, other studies showed that SGLT2i reduced EAT thickness in patients with and without diabetes and cardiovascular diseases.^29^, ^30^, ^31^

Although SGLT2i have been demonstrated to reduce EAT in patients with diabetes and obesity, their effects on EAT in HF across the ejection fraction spectrum remain unclear. Furthermore, no study has investigated the relationship between SGLT2i‐induced EAT changes and both inflammatory markers and cardiac magnetic resonance (CMR) parameters in patients with HFrEF and HFpEF. Since EAT contributes to inflammation and mechanical impairment of the heart, its reduction could play a beneficial role in improving cardiac structure and outcomes.

Therefore, this study aims to evaluate the impact of dapagliflozin on EAT in HFrEF and HFpEF using CMR data from the REFORM and DAPA‐LVH trials. Additionally, we will assess the associations between EAT changes, functional CMR parameters, and inflammatory markers.

## 2 METHODS

### 2.1 Study cohort

This analysis used combined data from two single‐centre, double‐blind, placebo‐controlled randomised clinical trials (RCTs) conducted in the Tayside region of Scotland: the REFORM trial (NCT02226510) and the DAPA‐LVH trial (NCT02956811). Briefly, both trials examined the effects of 10 mg dapagliflozin once daily in patients with T2D and heart failure phenotypes.

The REFORM trial included 56 participants with T2D and heart failure (stage C heart failure) (HF). Recruitment occurred between March 2015 and August 2016. Patients were identified through the Scottish Health Research Register (SHARE), Scottish Primary Care Research Network (SPCRN), Scottish Diabetes Research Network, Generation Scotland Database, and the Wellcome Study Database.

The DAPA‐LVH trial recruited 66 participants with T2D identified to have left ventricular hypertrophy (stage B heart failure) (LVH). Participants were recruited between February 2017 and May 2018 via research databases, hospital records, and local general practices.

In both trials, participants were randomised in a 1:1 ratio to receive 10 mg dapagliflozin once daily or placebo as standard care and were followed for 12 months (Figures S1 and S2).^23^, ^24^

### 2.2 Epicardial adipose tissue measurement

Cardiac magnetic resonance (CMR) imaging was performed according to standardised protocols as previously described in the REFORM and DAPA‐LVH protocols.^23^, ^24^ EAT quantification was conducted using the following methodology:

#### 2.2.1 Image acquisition and selection

ECG‐gated cine images were obtained using a TrueFISP sequence during a single breath‐hold. The long‐axis 4‐chamber view was selected as the standard plane for EAT measurement. After careful examination of all cardiac phases, the end‐diastolic frame was chosen for analysis.

#### 2.2.2 EAT identification and measurement

EAT was analysed in 96 of the 122 patients (49 from the dapagliflozin group and 47 from the placebo group). The main cause for exclusion was poor image quality in 17 patients caused by cardiac devices that generated artefacts in the images, and 9 patients were not available due to death or incomplete study.

EAT was defined as the high‐intensity fat located between the outermost aspect of the lower intensity myocardium and the low signal intensity visceral layer of the pericardium, as described previously.^32^, ^33^ Image analysis was performed offline using widely available open‐source Horos DICOM viewer.^34^, ^35^

**FIGURE 1:**
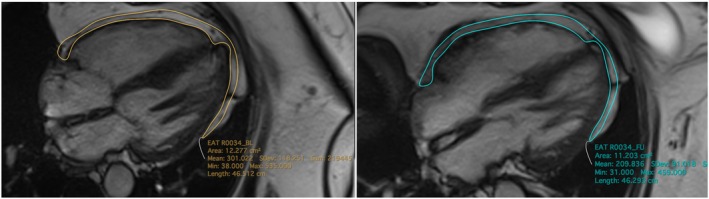
The 4 chambers CMR, EAT baseline measurement (left) and follow‐up measurement (right).

On the selected end‐diastolic 4‐chamber view image, the EAT area was delineated using the freehand region of interest (ROI) tool. Where possible, a single contiguous ROI was used to outline the EAT. In cases where there was no contiguous fat between two deposits, multiple ROIs were contoured and summated to calculate the total EAT area. The software automatically calculated the EAT area in square centimetres (cm^2^) based on the number of pixels within the outlined ROI(s) (Figure 1).

#### 2.2.3 Reliability measurement

To ensure measurement reliability, a second observer (KH) randomly checked and repeated measurements on a subset of images. Inter‐observer variability was evaluated by comparing repeated measurements, and agreement was quantified using the intraclass correlation coefficient (ICC). The analysis demonstrated excellent reproducibility, overall ICC was 0.92 (95% CI: 0.81–0.97) (Table S1).

### 2.3 Analysis of inflammatory markers

For both trials, blood samples were taken at baseline and a year later. Centrifugation for 10 min at 3000 rpm was used to segregate the plasma from these samples, which was then divided into aliquots and stored at −80°C. Plasma CRP levels were determined using the Luminex Human Discovery Assay (1‐Plex) LXSAHM‐01, and R&D Systems Luminex kits were used to measure the TNF, IL‐1, IL‐6, and IL‐10. All measurements were performed in duplicate, and all experimental methods were performed in accordance with the manufacturer's instructions.

### 2.4 Statistical analysis

All statistical analyses were performed with SPSS version 29 (IBM SPSS Inc., USA). Continuous variables are presented as mean ± standard deviation for normally distributed continuous variables, and median (interquartile range) for non‐normally distributed continuous variables. Categorical variables are described in terms of frequencies and percentages. Between‐group comparisons were conducted using independent Student *t* tests for normally distributed variables, while Mann–Whitney *U* tests were used for non‐normally distributed variables. ANCOVA (analysis of covariance) was carried out to assess the impact of dapagliflozin on EAT, cytokine levels, and functional cardiac parameters in comparison with placebo, adjusting for baseline values, age, sex, and BMI. To account for potential differences between the REFORM and DAPA‐LVH trial cohorts, trial group was included as a fixed factor in the ANCOVA model. Additionally, a treatment‐by‐trial interaction term was tested to evaluate whether the effect of dapagliflozin on EAT change differed between trials. The model also adjusted for baseline EAT, age, sex, and BMI. The association between alterations in EAT and other cardiac parameters and inflammation markers was also assessed using the Spearman correlation coefficient. Univariable linear regression analyses were first performed to explore associations between EAT and clinical, inflammatory, and cardiac parameters. Afterward, multivariable regression models were performed to evaluate independent associations with EAT at both baseline and follow‐up.

## 3 RESULTS

No significant differences existed at baseline between the dapagliflozin and placebo groups in terms of baseline demographics, including sex distribution (*p* = 0.84) and age (*p* = 0.26). There were no significant differences in blood pressure, heart rate, and BMI.

EAT thickness did not differ between groups (*p* = 0.37). Inflammatory markers, including CRP, IL‐1, IL‐6, IL‐10, and TNF, at baseline did not differ significantly (CRP: *p* = 0.29, IL‐1: *p* = 0.51, IL‐6: *p* = 0.18, IL‐10: *p* = 0.76). CMR parameters, including left ventricular ejection fraction, end‐diastolic volume, end‐systolic volume, and left ventricular mass, were comparable (LVEF: *p* = 0.66, EDV: *p* = 0.79, ESV: *p* = 0.8, LV‐mass: *p* = 0.58) (Table 1).

**TABLE 1:** Baseline characteristics.

Variables	Total cohort (*N* = 96); DAPA‐lvh (*N* = 49); REFORM (*N* = 47)	Dapagliflozin (*N* = 49); DAPA‐lvh (*N* = 25); REFORM (*N* = 24)	Placebo (*N* = 47); DAPA‐lvh (*N* = 24); REFORM (*N* = 23)	*p*‐value
HFpEF stage (B)	49 (51%)	25 (51%)	24 (49%)	—
HFrEF stage (C)	47 (49%)	24 (51.1%)	23 (48.9%)	—
Male	73 (62.9%)	37 (63.8%)	36 (62.1%)	0.84
Age (years)	65.6 ± 7	64.7 ± 7.5	65.9 ± 6.6	0.26
SBP (mmHg)	132.4 ± 14.5	132.4 ± 13.2	131.9 ± 15.9	0.54
DBP (mmHg)	74.7 ± 8.4	75.2 ± 78.2	74.7 ± 9	0.23
HR (bpm)	76.8 ± 13.5	77.3 ± 13.1	77.2 ± 14.3	0.49
BMI (kg/m^2^)	31.9 (29.8, 34.8)	31.9 (29.9, 35.1)	33 (30.2, 35.1)	0.78
Obesity (BMI ≥30)	72 (75%)	35 (71.4%)	37 (78.7%)	—
EAT (cm^2^)	11.8 (10.4, 14.3)	11.7 (10.4, 14)	12.6 (10.6, 14.7)	0.37
CRP (mg/L)	1.5 (0.5, 3.5)	1.6 (0.5, 3.5)	1.6 (0.5, 4.3)	0.29
IL‐1 (pg/mL)	0.5 (0.3, 0.5)	0.4 (0.3, 0.5)	0.5 (0.3, 0.5)	0.51
IL‐6 (pg/mL)	1.2 (1.1, 1.4)	1.2 (1, 1.4)	1.3 (1.1, 17)	0.18
IL‐10 (pg/mL)	0.5 (0.4, 0.6)	0.5 (0.4, 0.6)	0.5 (0.3, 0.7)	0.76
TNF (pg/mL)	4.9 (2.8, 6.6)	4.4 (2.2, 6.4)	5.5 (3.5, 6.9)	0.16
BNP (pg/mL)	544.3 (197.7, 1504.9)	523.2 (170.8, 1278)	683.2 (206.7, 3302.1)	0.18
LVEF (%)	64 (47.1, 73)	65.5 (48.8, 72)	65 (48.1, 73.5)	0.66
EDV (mL)	133.5 (116.5, 171)	136.5 (120, 167.5)	133.3 (108.6, 169.9)	0.79
ESV (mL)	48 (33, 94.4)	48 (33.3, 91.7)	46 (32.1, 70.9)	0.8
LV‐mass (g)	99.5 (72.1, 126.3)	102 (71.7, 131.7)	101.5 (75.9, 132.6)	0.58

*Note*: Data presented as mean ± SD or median (quartile 1, quartile 3).

Abbreviations: BMI, body mass index; CRP, C‐reactive protein; DBP, diastolic blood pressure; EAT, epicardial adipose tissue; EDV, end‐diastolic volume (mL); EF, ejection fraction; ESV, end‐systolic volume; HR, heart rate; IL‐1, interleukin‐1 beta; IL‐10, interleukin‐10; IL‐6, interleukin‐6; NT‐proBNP, N‐terminal pro b‐type natriuretic peptide; SBP, systolic blood pressure; TNF, tumour necrosis factor‐alpha.

### 3.1 Baseline regression analyses for EAT

Univariable linear regression showed only that BMI, LV mass, IL‐1, EF, and age were significantly associated with EAT thickness (*p* < 0.05). These variables were entered into a multivariable linear regression model. None remained significant, but BMI showed a trend toward significance (*B* = 0.2, 95% CI: −0.02 to 0.3, *p* = 0.07) (Table 2).

**TABLE 2:** Univariable and multivariable linear regression analyses examining baseline predictors of EAT thickness.

Variable	Univariable *B* (95% CI)	*p*‐value	Multivariable *B* (95% CI)	*p*‐value
BMI	0.2 (0.03, 0.4)	0.01	0.2 (−0.02, 0.3)	0.07
LV mass	0.03 (0.01, 0.1)	0.005	0.02 (−0.01, 0.05)	0.16
IL‐1	4.1 (0.03, 8.1)	0.04	1.4 (−2.9, 5.7)	0.52
LVEF	0.05 (0.004, 0.1)	0.03	0.02 (−0.04, 0.1)	0.46
Age	−0.1 (−0.2, −0.004)	0.04	−0.1 (−0.2, 0.04)	0.2

### 3.2 Baseline correlation of inflammatory biomarkers and EAT

At baseline, IL‐1 (*r* = 0.26, *p* = 0.01) and IL‐10 (*r* = 0.23, *p* = 0.03) correlated positively and significantly with EAT, suggesting a modest association of EAT with inflammatory processes. Associations of CRP (*r* = 0.16, *p* = 0.13) and IL‐6 (*r* = 0.18, *p* = 0.09) showed weak, non‐significant associations with EAT, while TNF (*r* = 0.14, *p* = 0.20) had no notable correlation (Table S2).

### 3.3 Baseline correlation of CMR parameters and EAT

EAT was positively correlated with LV mass (*r* = 0.25, *p* = 0.01) and showed a borderline significant association with LVEF (*r* = 0.2, *p* = 0.05). However, no significant correlations were found between EAT and EDV (*r* = −0.04, *p* = 0.72) or ESV (*r* = −0.17, *p* = 0.10) at baseline (Table S3).

### 3.4 Change in EAT, HR, and BMI

**FIGURE 2:**
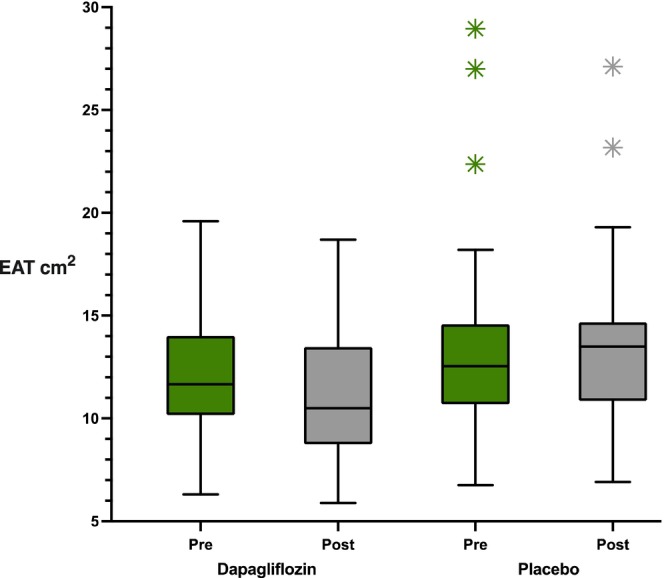
Comparison of median values pre‐ and post‐treatment EAT (cm^2^) in dapagliflozin vs. placebo groups, where asterisks in the figure refers to the outliers of EAT measurements in the placebo arm.

ANCOVA analysis demonstrated that dapagliflozin treatment resulted in a significant reduction in EAT independently of changes in BMI and other confounding factors compared with placebo. At follow‐up, EAT was 10.5 (8.9, 13.4) cm^2^ in the dapagliflozin group versus 13.4 (10.6, 14.9) cm^2^ in the placebo group, with a change of Δ = −1.2 ± 0.2 vs. 0.4 ± 0.2, *p* < 0.001, mean difference (MD) between groups was −1.5 cm^2^ (95% CI: −2 to 1). The treatment‐by‐trial interaction was not significant (*p* = 0.19), indicating that the effect of dapagliflozin on EAT reduction was consistent across both trials. BMI also decreased more markedly with dapagliflozin: 30.7 (27.8, 34.1) kg/m^2^ vs. 32.2 (30.9, 35.0) kg/m^2^ at follow‐up (Δ = −1.1 ± 0.2 vs. –0.2 ± 0.2, *p* < 0.001), MD between groups was −1 kg/m^2^ (95% CI: −1.5 to −0.5). Heart rate was significantly lower in the dapagliflozin group at follow‐up (77.8 ± 14.1 vs. 76.0 ± 13.6 bpm; Δ = −4.4 ± 1.5 vs. 0.7 ± 1.5, *p* = 0.01). There was no significant change in blood pressure between the dapagliflozin and placebo groups (Table 3; Figure 2).

**TABLE 3:** Follow‐up values and changes from baseline for EAT, inflammatory, and cardiac parameters in the dapagliflozin and placebo groups.

Variable	Dapagliflozin		Placebo		Mean difference (95% CI)	*p*‐value
	Follow‐up	Δ from baseline	Follow‐up	Δ from baseline		
SBP (mmHg)	129.5 ± 17.7	−3.6 ± 2	134.5 ± 13.6	1.7 ± 2.1	−5.2 (−11, 0.5)	0.07
DBP (mmHg)	75.1 ± 8	0.7 ± 1.2	73.1 ± 9.4	2.4 ± 1.3	−1.7 (−5.2, 1.8)	0.34
HR (bpm)	77.8 ± 14.1	−4.4 ± 1.5	76.0 ± 13.6	0.7 ± 1.5	−5.1 (−9.3, −0.9)	**0.01**
BMI (kg/m^2^)	30.7 (27.8, 34.1)	−1.1 ± 0.2	32.2 (30.9, 35)	−0.2 ± 0.2	−1 (−1.5, −0.5)	**<0.001**
EAT (cm^2^)	10.5 (8.9, 13.4)	−1.2 ± 0.2	13.4 (10.6, 14.9)	0.4 ± 0.2	−1.6 (−2, 1)	**<0.001**
CRP (mg/L)	1.6 (0.5, 5.1)	0.6 ± 0.4	2.2 (0.9, 3.7)	1.5 ± 0.5	−0.9 (−2.1, 0.4)	0.18
IL‐1 (pg/mL)	0.5 (0.2, 0.5)	−0.01 ± 0.15	0.5 (0.3, 0.5)	0.03 ± 0.11	−0.04 (−0.01, 0.07)	0.13
IL‐6 (pg/mL)	1.2 (0.9, 1.3)	−0.1 ± 0.1	1.2 (1.01, 1.5)	−0.1 ± 0.1	−0.01 (−0.3, 0.2)	0.94
IL‐10 (pg/mL)	0.6 (0.4, 0.7)	0.1 ± 0.02	0.66 (0.4, 0.8)	0.1 ± 0.02	−0.04 (−0.1, 0.02)	0.19
TNF (pg/mL)	4.9 (3.5, 6.7)	0.7 ± 0.2	6.1 (4.3, 7.03)	0.9 ± 0.2	−0.2 (−0.8, 0.5)	0.58
BNP (pg/mL)	427 (184.2, 1153.1)	−34.3 ± 219.9	568.9 (259.5, 2129.1)	−91.1 ± 226.1	56.8 (−571.3, 684.8)	0.85
LVEF (%)	66.9 (48.5, 75)	1.8 ± 0.8	63.2 (45.8, 71.9)	0.3 ± 0.8	1.5 (−0.6, 3.7)	0.16
EDV (mL)	139.4 (119.7, 160.9)	−2.9 ± 3.5	133 (110, 163.6)	−4.8 ± 3.6	1.9 (−8, 11.8)	0.70
ESV (mL)	45.5 (32.4, 79.1)	−4 ± 2.7	44.8 (33.3, 86)	−4.2 ± 2.8	0.2 (−7.4, 7.9)	0.95
LV‐mass (g)	96.6 (70.5, 128.1)	−3.5 ± 1.8	100 (75.2, 136.8)	1.6 ± 1.8	−5.1 (−10.1, −0.1)	**0.04**

*Note*: Data are presented as mean ± SD or median (IQR). Mean differences between groups are shown with 95% confidence intervals and *p*‐values. Bold values indicate statistically significant differences (*p* < 0.05).

### 3.5 Change in inflammatory markers

Changes from baseline in inflammatory and cardiac biomarkers over 12 months were compared between the dapagliflozin and placebo groups. CRP increased less in the dapagliflozin arm compared with placebo (Δ = 0.6 ± 0.4 mg/L vs. Δ = 1.5 ± 0.5 mg/L; MD: −0.9 mg/L, 95% CI: −2.1 to 0.4; *p* = 0.18). IL‐1 decreased slightly with dapagliflozin while increasing with placebo (Δ = −0.01 ± 15 pg/mL vs. Δ = 5.2 ± 3.6 pg/mL; MD: −5.3 pg/mL, 95% CI: −15.1 to 4.5; *p* = 0.28). Both groups showed similar changes in IL‐6 (Δ = −0.1 ± 0.1 pg/mL; MD: −0.01 pg/mL, 95% CI: −0.3 to 0.2; *p* = 0.94), IL‐10 (Δ = 0.1 ± 0.02 pg/mL; MD: −0.04 pg/mL, 95% CI: −0.1 to 0.02; *p* = 0.19), and TNF (Δ = 0.7 ± 0.2 pg/mL vs. Δ = 0.9 ± 0.2 pg/mL; MD: −0.2 pg/mL, 95% CI: −0.8 to 0.5; *p* = 0.58). BNP decreased in both groups (Δ = −34.3 ± 219.9 pg/mL vs. Δ = −91.1 ± 226.1 pg/mL; mean difference: 56.8 pg/mL, 95% CI: −571.3 to 684.8; *p* = 0.85) (Table 3).

### 3.6 Changes in CMR parameters

Dapagliflozin modestly improved ejection fraction (66.9% vs. 63.2%; Δ = 1.81% ± 0.75% vs. 0.30% ± 0.78%), although the difference was not statistically significant (*p* = 0.165).

No significant differences were observed in end‐diastolic volume (EDV: 139.4 vs. 133.0 mL, *p* = 0.705) or end‐systolic volume (ESV: 45.5 vs. 44.8 mL, *p* = 0.955), with similar reductions from baseline in both groups.

Left ventricular mass (LV‐mass) was lower at follow‐up in the dapagliflozin group (96.6 g) compared with placebo (100.0 g), with a significant between‐group difference in change (−3.53 ± 1.77 g vs. 1.57 ± 1.83 g, *p* = 0.048). Global longitudinal strain (GLS), available only in the DAPA‐lvh, showed a non‐significant trend to improvement with dapagliflozin versus placebo (Δ = −1.78% vs. −0.45%; between‐group difference −1.33% [95% CI −2.88 to +0.22], *p* = 0.09) (Table S6).

### 3.7 Follow up regression analyses for EAT

In the univariable regression analysis, the only variable that was found to be significantly associated with a change in EAT was the change in IL‐1 (*B* = 0.016, *p* = 0.007). Since no other variables were significant, we updated the multivariable model to include change in IL‐1, BMI, HR, and SBP/DBP as clinically relevant covariates to adjust for potential confounding effects on EAT. In this adjusted model, the association between change in IL‐1 and change in EAT remained statistically significant (*B* = 0.016, *p* = 0.004), although none of the other factors approached significance.

### 3.8 Follow up correlation of inflammatory biomarkers and EAT

There were no significant correlations observed between differences in inflammatory markers and EAT decrease over 12 months (CRP: *r* = 0.087, *p* = 0.414; IL‐1: *r* = 0.068, *p* = 0.523; IL‐6: *r* = 0.138, *p* = 0.198; IL‐10: *r* = 0.085, *p* = 0.420; TNF: *r* = 0.034, *p* = 0.746). These findings imply that while EAT is correlated to certain inflammatory markers at baseline, changes in EAT following dapagliflozin treatment do not directly correlate with inflammatory marker alterations (Table S7).

### 3.9 Follow up correlation of CMR parameters and EAT

Over 12 months, no significant associations were observed between changes in EAT and structural cardiac parameters, including LVEF (*r* = −0.09, *p* = 0.34), EDV (*r* = −0.11, *p* = 0.29), ESV (*r* = 0.04, *p* = 0.66), and LV mass (*r* = 0.01, *p* = 0.96) (Table S8).

## 4 DISCUSSION

The key novel approach in our study was the evaluation of EAT across the heart failure spectrum in patients with diabetes. Our findings demonstrated that dapagliflozin treatment significantly reduced EAT thickness, BMI, and LV mass in patients with heart failure and type 2 diabetes. However, despite significant associations between EAT and inflammatory markers at baseline, and the reduction in EAT after treatment, dapagliflozin did not significantly alter levels of inflammatory markers, including CRP, IL‐1, IL‐6, IL‐10, and TNF, over 12 months. In addition to that, we did not observe significant correlations between changes in EAT and inflammatory markers or cardiac functional parameters.

EAT has emerged as a key contributor to the pathophysiology of heart failure, playing a significant role in both HFpEF and HFrEF.^9^, ^10^ Its accumulation has been repeatedly linked with structural and functional cardiac abnormalities, including diastolic dysfunction, left ventricular hypertrophy, and atrial enlargement, particularly in HFpEF.^9^, ^11^, ^12^ These observations imply mechanisms that may account for the increased risk of heart failure associated with greater EAT volume.

The significant reduction in EAT thickness with dapagliflozin in our cohort (−1.16 ± 0.18 cm^2^ vs. +0.36 ± 0.19 cm^2^ with placebo, *p* < 0.001) strengthens evidence that SGLT2 inhibitors effectively reduce fat deposits. This finding aligns with previous studies showing a similar EAT reduction in type 2 diabetes and obesity patients receiving dapagliflozin.^29^, ^36^ SGLT2 inhibitors likely achieve this effect through enhanced insulin sensitivity, improved glycaemic control, and favourable modulation of inflammatory and metabolic signalling within adipose tissues, although the precise inflammatory pathways involved remain incompletely understood. Masson et al.'s (2021) meta‐analysis confirmed that SGLT2i therapy significantly reduces EAT across various clinical contexts. As EAT is recognised as an active pro‐inflammatory depot implicated in cardiovascular disease pathogenesis, this reduction may partially explain the cardioprotective effects observed in major SGLT2i trials.^27^ These results highlight the dual metabolic and structural benefits of dapagliflozin and support incorporating EAT as a relevant imaging biomarker in future cardiometabolic research.

Similarly, several studies have evaluated the effects of different SGLT2 inhibitors including empagliflozin, dapagliflozin, luseogliflozin, and ipragliflozin EAT in patients with T2DM, both with and without obesity. These agents consistently demonstrated significant reductions in EAT thickness following treatment durations ranging from 3 to 6 months. For instance, empagliflozin significantly reduced EAT volume and improved myocardial energetics in the EMPACEF study, while dapagliflozin led to a marked decrease in EAT area in patients with T2DM and obesity.^37^, ^38^ In non‐obese patients with visceral adiposity, ipragliflozin also demonstrated reductions in EAT accumulation.^39^ Also, luseogliflozin has been shown to reduce EAT volume in Japanese patients with type 2 diabetes, in parallel reductions in body weight.^40^ Across these investigations, reductions in EAT were frequently accompanied by improvements in BMI, supporting the hypothesis that SGLT2 inhibitors exert a systemic effect on adipose tissue metabolism. This includes not only a decrease in visceral fat depots but potentially an inflammatory effect, collectively contributing to their cardiovascular protective benefits. Recent evidence also suggests that these benefits arise from a broader spectrum of mechanisms: their dual action on the heart and kidneys improves hemodynamic and slows renal decline,^41^ while they also enhance myocardial energy efficiency, limit fibrosis, improve endothelial function.^42^ In addition, SGLT2i modulate adipose biology by reducing pro‐inflammatory epicardial adipose tissue, and improving insulin sensitivity, collectively improving adipose‐derived inflammation and supporting coronary microvascular function.^43^

Importantly, other classes of cardiometabolic therapies may exert even greater effects on EAT. In particular, GLP‐1 receptor agonists (GLP‐1 RAs) have been suggested to provide stronger reductions than SGLT2 inhibitors, mainly through weight loss, improved metabolism, and direct effects on visceral fat depots including EAT.^44^

In addition to the effects on EAT and BMI, we also investigated the effects of dapagliflozin on LV mass, as several studies have shown that the SGLT2i (dapagliflozin, empagliflozin, etc.) can significantly decrease LV mass, which is consistent with our results.^24^, ^45^, ^46^, ^47^ Furthermore, it is established that weight loss therapies are correlated with significant reductions in LV mass.^48^ Taken together, these observations support the opinion that SGLT2 inhibitors may reduce LV mass by targeting both EAT and overall body weight, highlighting their potential to improve cardiac structure and function in patients with heart failure and type 2 diabetes.

In parallel with imaging markers of remodelling, we investigated myocardial function via GLS in the DAPA‐lvh cohort. After baseline adjustment, dapagliflozin showed a non‐significant trend to improve GLS versus placebo. Moreover, higher EAT tracked with less negative GLS at baseline and follow‐up, but these associations were not significant. This overall pattern is consistent with prior T2D evidence that greater EAT thickness marks subclinical systolic dysfunction and reduced exercise capacity, and with data showing empagliflozin improves LV‐GLS primarily when baseline GLS is impaired (<16.5%).^49^, ^50^

Thus, SGLT2 inhibitors play an essential role in reducing visceral adiposity, particularly EAT, a known risk factor for cardiovascular disease. The observed reductions in heart rate and LV mass suggest possible cardioprotective effects, even in the absence of significant changes in inflammation or other structural cardiac parameters. These results support the growing evidence that SGLT2 inhibitors provide cardiovascular benefits beyond glucose‐lowering effects.

There are several limitations that should be recognised. First, the 12‐month follow‐up period may have been inadequate to fully capture the impact of EAT reduction on cardiac function and remodelling. Second, some patients were excluded for poor image quality and for the unavailability of some blood samples, which may have affected the accuracy of EAT measurements and the evaluation of EAT correlation with inflammatory markers. Also, no T1‐mapping ECV data were acquired, restricting fibrosis assessment and interpretation of structural remodelling. Moreover, although based on randomised controlled trial data, this was a retrospective pooled analysis of two separate trials with differing inclusion criteria: DAPA‐lvh enrolling patients with stage B HF (LV hypertrophy) and REFORM enrolling those with stage C HF. Finally, this study was limited to patients with T2D, and our findings may not be generalisable to HF patients without diabetes.

## 5 CONCLUSION

In summary, dapagliflozin significantly reduces EAT volume, BMI, and LV mass in patients with HF and T2D. However, these reductions do not correlate with changes in inflammatory markers or cardiac function, suggesting that SGLT2 inhibitors exert their beneficial effects on cardiac structure through mechanisms independent of systemic inflammation. These findings provide further support for the cardiometabolic benefits of SGLT2 inhibitors in HF management, though longer‐term studies are needed to clarify the clinical significance of EAT regression and its impact on HF outcomes.

## CONFLICT OF INTEREST STATEMENT

The authors declare no conflicts of interest.

## PEER REVIEW

The peer review history for this article is available at https://www.webofscience.com/api/gateway/wos/peer‐review/10.1111/dom.70164.

## Supporting information


**Data S1.** Supporting Information.

## Data Availability

Data available on request from the authors.
