# Supplementary material for: Dapagliflozin reduces epicardial adipose tissue in patients with heart failure and type 2 diabetes
Source: Diabetes Obes Metab. 2025 Oct 6;27(12):7561–9. doi: 10.1111/dom.70164 (PMC12587262; doi:10.1111/dom.70164)
Supplement: Supplementary file 1 — Data S1. Supporting Information. [file DOM-27-7561-s001.docx]

| **Study** | **EAT thickness 1^st^ observer** | **EAT thickness 2^nd^ observer** | **ICC** | **95% CI** |
| --- | --- | --- | --- | --- |
| **REFORM** | 13.1 ± 3.8 | 12.7 ± 3.6 | 0.95 | 0.82 − 0.97 |
| **DAPA-lvh** | 13.4 ± 2.1 | 13 ± 1.9 | 0.86 | 0.74 − 0.96 |
| **Both studies** | 13.3 ± 3 | 12.9 ± 2.9 | 0.92 | 0.81 − 0.97 |

**Table 1.** Inter-observer agreement for EAT thickness in REFORM and DAPA-LVH, expressed as ICC with 95% confidence intervals.

| **Variable** | **Baseline correlation with EAT (r)** | **P-value** |
| --- | --- | --- |
| **CRP** | 0.16 | 0.13 |
| **IL-1** | 0.26 | **0.01** |
| **IL-6** | 0.18 | 0.09 |
| **IL-10** | 0.23 | **0.03** |
| **TNF** | 0.14 | 0.20 |

| **Table 2.** Baseline correlations between inflammatory biomarkers and EAT |
| --- |

| **Variable** | **Baseline correlation with EAT (r)** | **P-value** |
| --- | --- | --- |
| **LVEF** | 0.2 | 0.05 |
| **EDV** | -0.04 | 0.72 |
| **ESV** | -0.17 | 0.1 |
| **LV_mass** | 0.25 | **0.01** |

| **Table 3.** Baseline correlations between EAT and structural cardiac parameters |
| --- |

| **Variable** | **Total cohort** | **Dapagliflozin** | **Placebo** |
| --- | --- | --- | --- |
| **DAPA-LVH study** | | | |
| HbA1c (mmol/mol) | 61.7 ± 10.5 | 62.6 ± 10.7 | 60.8 ± 10.3 |
| Metformin N=49 | 49 (100%) | 25 (100%) | 24 (100%) |
| Insulin N=14 | 14 (23.3%) | 7 (24.1%) | 7 (22.6%) |
| **REFORM study** | | | |
| HbA1c (mmol/mol) | 60.9 ± 17.1 | 63.0 ± 17.8 | 58.6 ± 16.4 |
| Metformin N=24 | 24 (55.4%) | 14 (60.7%) | 10 (49%) |
| Insulin N=15 | 15 (28.6%) | 6 (21.1%) | 9 (35.6%) |

**Table 4**. HbA1c values and glucose-lowering therapies at baseline

| **Variables** | **DAPA-LVH Cohort (N=35)** | **Dapagliflozin (N=17)** | **Placebo (N=18)** | **p-value** |
| --- | --- | --- | --- | --- |
| GLS (%) | −17.7 (−19, −16) | −17.8 (−19.6, −15.6) | −17.7 (−19, −16) | 0.48 |

Table 5. Baseline characteristics in the DAPA-LVH cohort. GLS, global longitudinal strain. Data are presented as median (quartile 1, quartile 3).

| **Variable** | **Dapagliflozin** | | **Placebo** | | **Mean difference (95% CI)** | **P-value** |
| --- | --- | --- | --- | --- | --- | --- |
|  | **Follow-up** | **Δ from baseline** | **Follow-up** | **Δ from baseline** |  |  |
| GLS (%) | −18.2 (−22.2, −16.9) | −1.78 (−2.82, −0.74) | −18.5 (−19.8, −17.0) | −0.45 (−1.58, +0.69) | +1.33 (−0.22, +2.88) | 0.09 |

Table 6. Follow-up values and changes from baseline for GLS with Mean difference (95% CI) in the DAPA-LVH cohort.

| **Variable** | **Correlation with change in EAT at 12 months (*r*)** | **P-value** |
| --- | --- | --- |
| **CRP** | 0.09 | 0.41 |
| **IL-1** | 0.07 | 0.52 |
| **IL-6** | 0.14 | 0.19 |
| **IL-10** | 0.09 | 0.42 |
| **TNF** | 0.03 | 0.74 |

| **Table 7.** Correlations between changes in inflammatory biomarkers and change in EAT at 12 months |
| --- |

| **Variable** | **Correlation with change in EAT (r)** | **P-value** |
| --- | --- | --- |
| **LVEF** | -0.09 | 0.34 |
| **EDV** | -0.11 | 0.29 |
| **ESV** | 0.04 | 0.66 |
| **LV_mass** | 0.005 | 0.96 |

| **Table 8.** Correlations between changes in EAT and structural cardiac parameters |
| --- |

| **Variable** | **Univariable B (95% CI)** | **P-value** |
| --- | --- | --- |
| **Absolute GLS (baseline)** | **−0.37** (−1.17, +0.43) | 0.355 |
| **Absolute GLS (follow-up)** | **−0.24** (−0.88, +0.41) | 0.465 |

Table 9. Association between EAT and GLS at baseline and follow-up (univariable regressions; DAPA-LVH only)


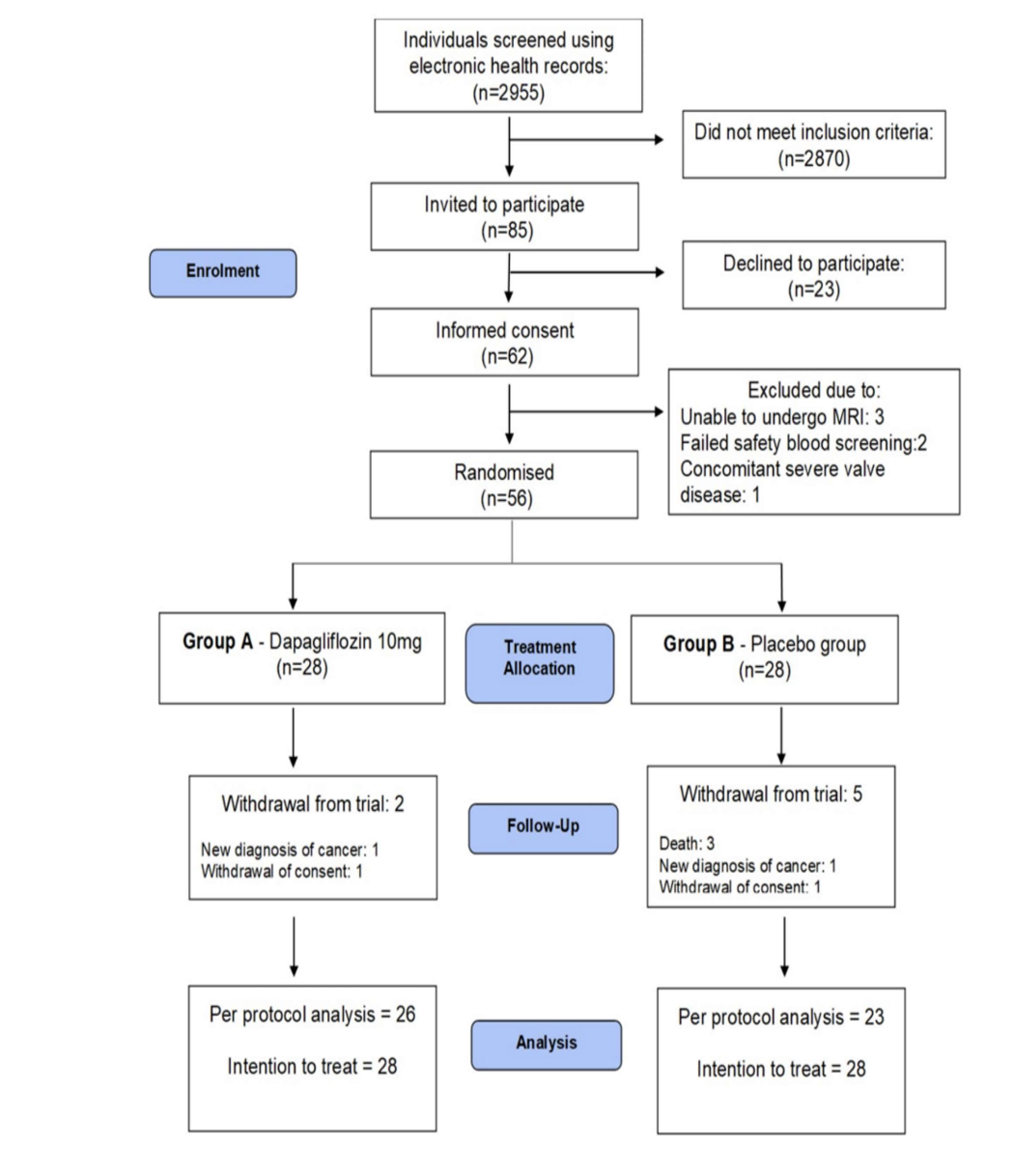


**Figure 1**. Study design diagram of REFORM trial.


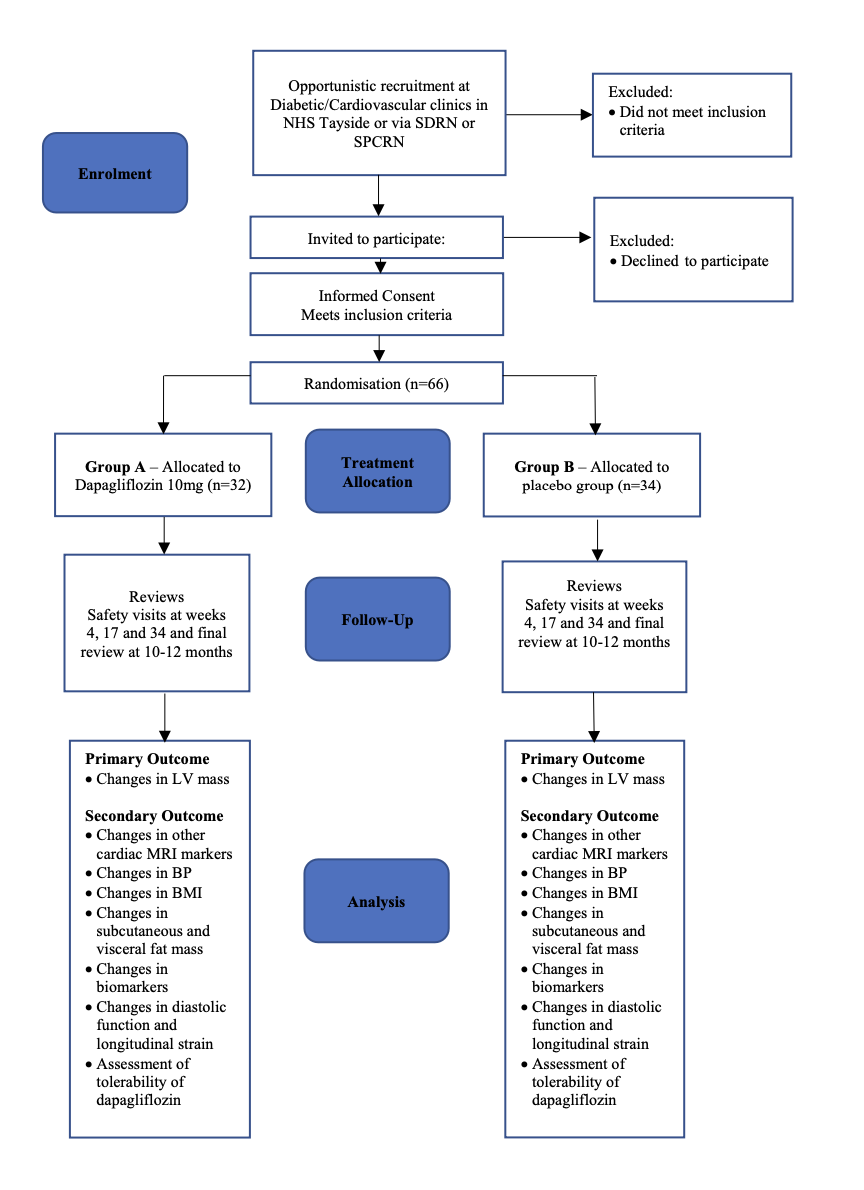


**Figure 2**. Study design diagram of DAPA-lvh trial
